# Neuroplasticity of children in autism spectrum disorder

**DOI:** 10.3389/fpsyt.2024.1362288

**Published:** 2024-04-25

**Authors:** Zilin Chen, Xu Wang, Si Zhang, Fei Han

**Affiliations:** ^1^ Department of Pediatrics, Guang’anmen Hospital, China Academy of Traditional Chinese Medicine, Beijing, China; ^2^ Experiment Center of Medical Innovation, The First Hospital of Hunan University of Chinese Medicine, Changsha, Hunan, China

**Keywords:** autism spectrum disorder, neuroplasticity, genetics, neural pathways, neuroinflammation, immunity

## Abstract

Autism spectrum disorder (ASD) is a neurodevelopmental disorder that encompasses a range of symptoms including difficulties in verbal communication, social interaction, limited interests, and repetitive behaviors. Neuroplasticity refers to the structural and functional changes that occur in the nervous system to adapt and respond to changes in the external environment. In simpler terms, it is the brain’s ability to learn and adapt to new environments. However, individuals with ASD exhibit abnormal neuroplasticity, which impacts information processing, sensory processing, and social cognition, leading to the manifestation of corresponding symptoms. This paper aims to review the current research progress on ASD neuroplasticity, focusing on genetics, environment, neural pathways, neuroinflammation, and immunity. The findings will provide a theoretical foundation and insights for intervention and treatment in pediatric fields related to ASD.

## 1 Introduction

Autism spectrum disorder (ASD), also known as autism or autistic disorder, is most commonly diagnosed in children. It encompasses a group of genetically heterogeneous neurobehavioral disorders characterized by impairments in three core areas: communication, social interaction, and stereotypical repetitive behaviors. Epidemiological data suggests ASD affects approximately 2% of children, with a male-to-female ratio of 4:1, and heritability estimates ranging from 70% to 90% (1). Globally, the prevalence of ASD in children under 5 years old decreased from 1990 to 2019, but prevalence and disability-adjusted life year (DALY) rates increased (2). Children with developmental delays, intellectual disabilities, or epilepsy exhibit longer and more varied stereotypes (3). ASD development is influenced by a variety of factors, including complex interactions between genetic and environmental factors, as well as functional and structural abnormalities in neurodevelopment (4).

Neuroplasticity refers to adaptive changes in the structure and function of the nervous system that allow people to learn, remember, and adapt to new environments through experience. Plasticity is achieved primarily through regulation of genetic, molecular, and cellular mechanisms enabling gain or loss of behavior or function by influencing synaptic connections and neural circuit formation. Abnormalities in neuroplasticity have been found in brains of patients with ASD, potentially leading to abnormal neuronal connectivity and circuit formation, affecting information transmission and coordination between brain regions (5). This plasticity is a complex process with increased sensitivity during fetal and infant brain development and to a lesser extent during adolescence and adulthood (6). This review focuses on the important role of neuroplasticity in ASD in terms of genetics, environment, neural pathways, neuroinflammation, and immunity to inform the design, timing, and sequencing of neuromodulatory interventions for clinical research to enhance and optimize their translational application in childhood brain disorders.

## 2 Genetic and environmental factors

### 2.1 Chromosomal abnormalities

ASD has a clear genetic predisposition, and although heritability estimates for ASD range from 70% to 90%, the molecular diagnosis rate is lower than expected (7). The New Jersey Language and Autism Genetics Study (NJLAGS) investigated more than 100 families with at least one member with ASD (8). Identical twins have a higher concordance of ASD symptoms compared to dizygotic twins (9). Up to 40% of children with ASD are diagnosed with genetic syndromes or chromosomal abnormalities, including small DNA deletions or duplications, genetic variants, and metabolic disorders with mitochondrial dysfunction, among others, all suggesting a genetic influence on ASD (10). In particular, chromosomal abnormalities are important in the pathogenesis of ASD, and the relative telomere length of chromosomes has a sexually dimorphic pattern in ASD, with boys with ASD having significantly shorter relative telomere lengths than typically developing controls and paired siblings (11). Numerous studies show many diseases resembling ASD-like phenotypes often involve chromosome 15q11-q13 segments (12). Deletions and duplications of chromosomes 15q11.2, 15q13.3, 16p11.2, 22q11.2, and BP1-BP2 are among the common genetic causes of ASD (1, 13).

### 2.2 Genetic mutations

The pattern of inheritance of ASD involves numerous rare gene mutations, many clustered in nervous system maturation, including neurogenesis, axonal development, and synapse formation. These play a critical role in normal neuroplasticity development (14). On chromosome 17q12, the LHX1 gene is associated with neurodevelopmental abnormalities and plays a role in GABA neuron migration/differentiation, affecting interneuron development/survival (15). In animal experiments, CSMD3 gene deletion resulted in abnormal dendritogenesis of cerebellar Purkinje cells in mice, producing typical ASD symptoms and motor dysfunction (16). Genetic variants in the γ-aminobutyric acid receptor subunit gene, which encodes the neurotransmitter receptor, also contribute to the development of ASD (17). In addition, ASD patients with mutations in GRIN2B, SHANK3, and UBTF genes exhibit hyperexcitability and early cortical neuron maturation, characterized by increased sodium currents, larger/faster excitatory postsynaptic currents, and more action potentials during early neuron development (18). Most ASD-related genes determine the function of the nervous system and affect certain protein activities and cellular functions in genetic information propagation, such as disruption of protein localization on the mitotic spindle, cell cycle arrest, DNA damage and cell death (19). In terms of single genes, haploinsufficiency in KMT2E (20), Taok1 (21), PLPPR4 (22), ZDHHC15 (23), ASH1L (24), and Kdm6b (25)affects neurological development and produces ASD symptoms. The CAPRIN1 gene is critical for synaptic plasticity, encoding a ubiquitous protein regulating mRNA transport and translation. CAPRIN1 haploinsufficiency leads to neuronal tissue destruction, impaired calcium signaling with increased oxidative stress, and developmental/functional deficits of the neuronal network, including language deficits, attention deficit hyperactivity disorder, and ASD (26). Thus, some potent neuroplasticity modulators have the potential to treat ASD. Haploinsufficiency of the SHANK3 gene on chromosome 3q22.13 usually results in severe deficits in motor behavior, sensory processing, language, and cognitive function. An IGFBP2 mimetic peptide fragment, JB2, directly binds to dendrites and synapses, rescuing these deficits and facilitating neuronal culturing and repair of synaptic functional plasticity (27).

### 2.3 Environmental factors

**Figure 1:**
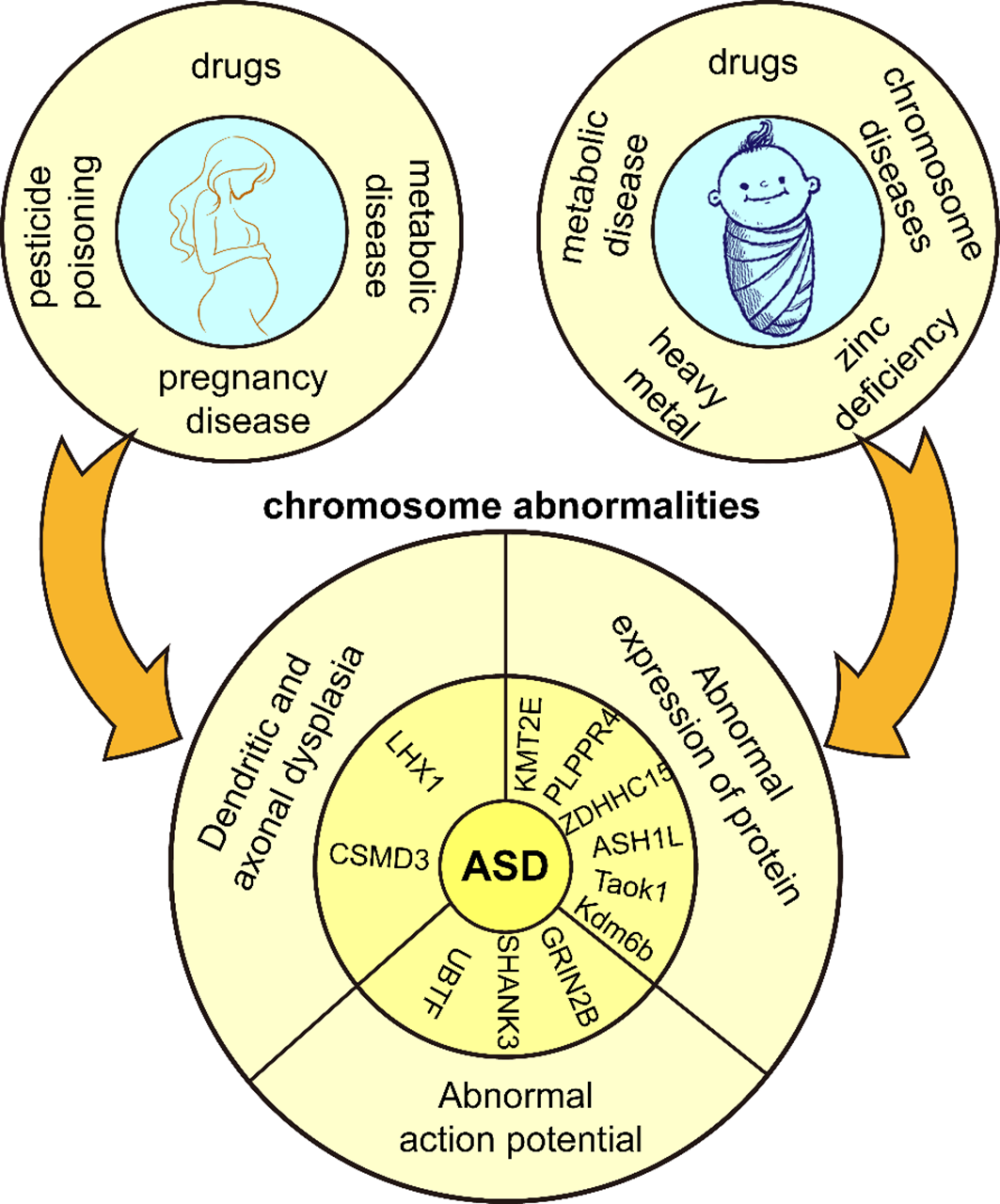
Genetic and environmental factors of ASD. Adverse external environmental factors exposed to mothers and children lead to chromosomal abnormalities in children and eventually ASD.

Environmental factors play an important role in the development of ASD (28). The etiology of ASD often involves a complex interaction between genetic and environmental factors. Some mutations are not considered to be the direct cause of the development of ASD, but rather their mutation is induced by environmental factors. Maternal dietary habits, prenatal use of pharmacologic agents (29), diabetes (30), obesity (31), asthma (32), and deficiencies in important nutritional factors increase gene mutation rates (33). Fetal hypoxia or asphyxia, mother’s history of multiple spontaneous abortions (34), bleeding during pregnancy and complications during labor and delivery (35) also increase ASD risk in offspring. It is worth mentioning that parental education level can impact the symptoms of ASD. Parents with higher education tend to pay more attention to prenatal care, family environment, and parenting styles, which can reduce the likelihood of risk factors triggering the disorder and facilitate early detection and intervention in the early stages of the disorder (36). Additionally, pesticide poisoning also contributes to the development of ASD. Chlorpyrifos is a widely used organophosphorus pesticide that induces dysfunctional synaptic plasticity in rat hippocampal neurons (37). Maternal organochlorine pesticide exposure increases the risk rate of ASD in the offspring (38). The incidence of pesticide poisoning is two times higher in males than in females, and the number of ASDs and disability-adjusted life years (DALYs) are four times higher in males than in females, and pesticide poisoning may be a contributing factor to gender differences in ASDs (39). Macronutrients and micronutrients can harm human health when outside normal ranges. Toxic substances produce chemical effects in the body, and overexposure to toxic heavy metals or inadequate essential metal intake during fetal and infant life correlate positively with ASD prevalence (40). Children with ASD have higher concentrations of chromium (Cr), mercury (Hg), arsenic (As), manganese (Mn), copper (Cu), and aluminum (Al) in their blood compared to neurotypical children, often accompanied by zinc deficiencies, which is a major cause of social difficulties and language skill deficits (41); Copper induces neuronal degeneration and oxidative damage, disrupts synaptic plasticity and inhibits the CREB/BDNF pathway, exacerbating learning and memory deficits (42); Zinc deficiency may lead to decreased cognitive and learning abilities and increased oxidative stress (43). Some metabolic and chromosomal disorders such as Down syndrome, phenylketonuria, Rett syndrome, and fragile X syndrome are also common genetic causes of ASD (44). In summary, ASD occurs in response to unfavorable internal and external factors during a critical period of central nervous system development, when neural structure and function are impaired, and consequently, associated symptoms (**Figure 1**). However, the mechanisms by which these factors influence neuroplasticity have not been adequately supported by research and need to be further explored.

### 2.4 Sexual dimorphism

Many researchers have observed increasing gender differentiation in ASD. Some ASD-associated miRNAs exhibit sexual dimorphism, such as miR-219 and miR-338, promoting oligodendrocyte differentiation, miR-125 involved in neuronal differentiation, and miR-488 associated with anxiety. Although their target genes of these miRNAs are significantly enriched in neural pathways in both sexes, less than one-third of the targets are shared between the sexes (45). As the main difference between males and females is in sex chromosomes and many gender-associated genes. Therefore, a pertinent question arises: does the X chromosome mitigate ASD prevalence, or does the Y chromosome elevate predisposition? For example, in Turner syndrome, the female phenotype has only one X chromosome and about 3% have ASD, 3 times the risk of the general population (46). In children with the XYY syndrome chromosome, about 14% have ASD (47). It can be hypothesized that the X chromosome may be a protective factor and the Y chromosome may be a risk factor for ASD. In a study of the female protective hypothesis, it was discovered that females with ASD who carried a higher number of ASD-associated risk alleles in the OXTR gene demonstrated enhanced functional connectivity between the nucleus accumbens. Females exhibit stronger functional connectivity between cortical regions involved in learning and motor control compared to males (48). Overexpression of eIF4E in microglia leads to higher synaptic density, neuroligins, and excitation-to-inhibition ratio in cortical neurons of male mice that exhibit ASD-like behaviors (49). These mice exhibit unique social deficits and reduced spine density in relevant neurons, possibly due to enhanced microglial phagocytosis leading to excessive pruning of synapses and disruption of neural circuits controlling social behavior (50). However, not all findings indicate that males exhibit more prominent ASD phenotypes. Research has shown that young girls diagnosed with ASD often have greater social communication deficits than young boys in toddlers and preschool children (51). Additionally, the functional impairment of some candidate risk genes for ASD, such as EPHB2, results in more repetitive behaviors, hyperactivity, and learning and memory deficits in models of female mice exhibiting typical ASD behaviours (52). The sexual dimorphism of ASD is influenced by various factors, but the primary mechanisms underlying its occurrence and effects require further in-depth research.

## 3 Neural pathways

### 3.1 Neurotransmitter abnormalities

**Figure 2:**
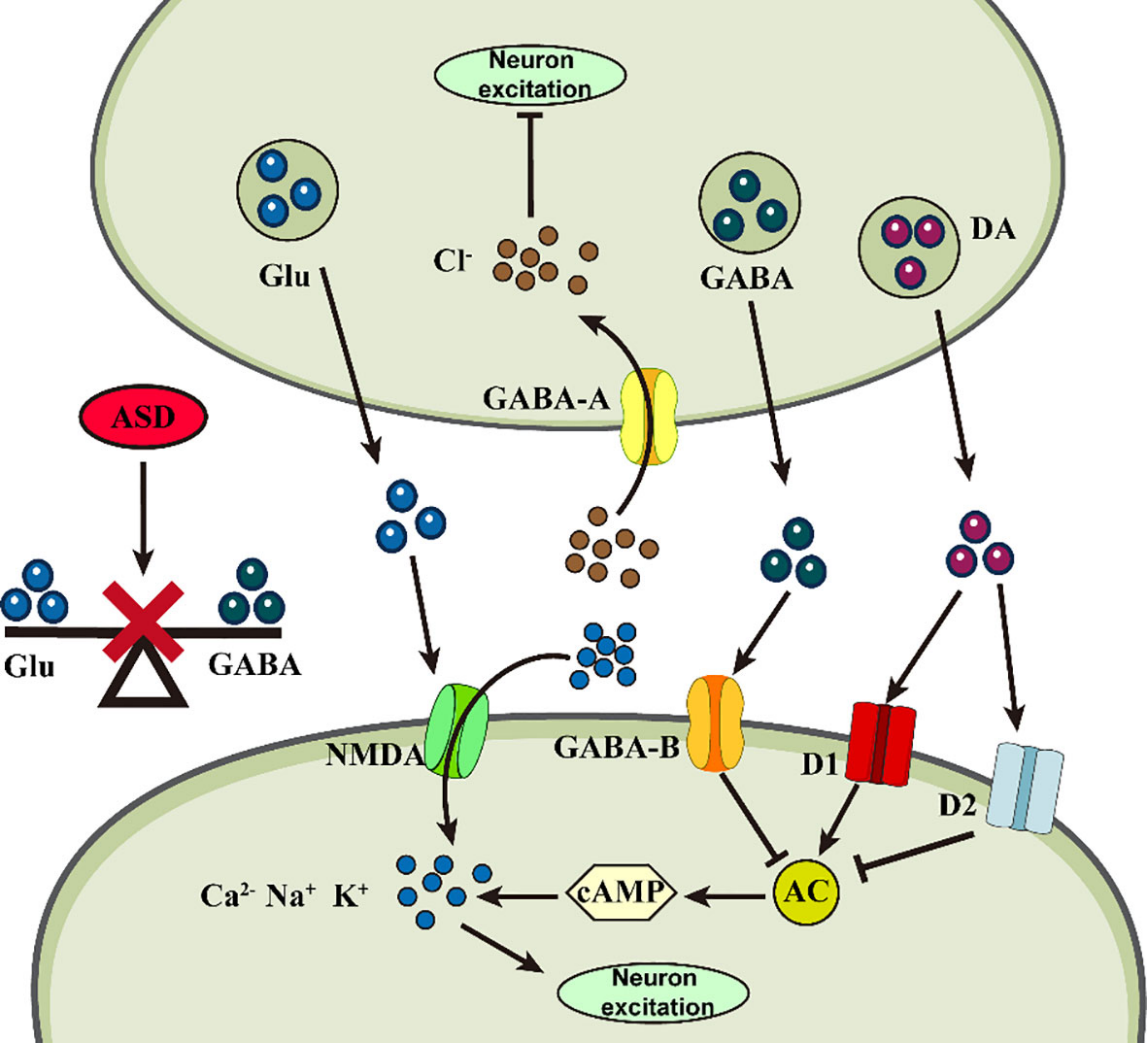
Abnormalities of neurotransmitters in ASD. Abnormal changes in different neurotransmitters at the synapse may impair neuronal plasticity, triggering ASD.

Neurotransmitters are chemicals that transmit signals between neurons, and an abnormal neurotransmitter system can lead to malfunctioning signaling between neurons, disrupting the process of neuroplasticity. The process of neuroplasticity involves various amino acids and their derivatives, including glutamate (Glu), glycline (Gly), γ-aminobutyric acid (GABA), 5-hydroxytryptamine (5-HT), dopamine (DA), and norepinephrine (NE), among others (**Figure 2**).

Glu is an excitatory neurotransmitter that not only regulates synapse formation, but also participates in astrocyte migration, differentiation, and apoptosis and is responsible for learning, memory, behavioral, motor, and sensory functions (53). Gly and Glu act as co-agonists to induce Glutamatergic N-methyl-D-aspartate (NMDA) excitatory potentials, resulting in enhanced neuroexcitotoxicity (54). GABA is an inhibitory neurotransmitter responsible for brain development, cognitive activity, and attention formation (55). All three amino acids are involved in maintaining homeostasis and balance in the nervous system. Abnormally high levels of Glu and abnormally low GABA receptor binding in the nervous system disrupt this balance, resulting in synaptic overstimulation and aberrant neuronal excitation, leading to cognitive impairment (56). Mean plasma Glu levels were significantly higher in children with ASD compared to the normal group and increased with increasing severity of ASD (57). However, GABA levels, which were also relatively increased, exhibited excitotoxicity in neurons, probably due to a decrease in the number of GABA receptors, the absence of an effective receptor uptake system, and the accumulation of GABA in the extracellular space to a sufficiently high level (58). The high-Gluergic state reflects an imbalance in the ASD brain that is excitatory rather than inhibitory (59). These findings can serve as biomarkers for clinical ASD diagnosis, as children’s developing brains are more susceptible to neuroexcitotoxic effects. Based on these theories, they may provide novel ideas for clinical ASD treatment. An NMDA receptor antagonist, memantine, has been found to modulate the disturbed relationship between Glutamine (Gln), Glu, and GABA and maintain nervous system homeostasis (60). A study noted that bumetanide can alter the synaptic excitatory-inhibitory (E-I) balance by potentiating GABA effects, thereby reducing ASD symptom severity in animal models (61). Icariin (ICA) could be a promising new therapeutic agent for ASD, attenuating disturbed E-I synaptic protein balance in model animals, suppressing neuroinflammation, and alleviating ASD symptoms by decreasing pro-inflammatory cytokine protein levels (62).

5-HT in the nervous system is responsible for synaptogenesis and regulation of neuronal activity and for the regulation of mood, memory and learning in the brain (63). 5-HT neurotransmission dysfunction and circuit dysregulation may underlie the behavioral abnormalities associated with ASD (64). Patients with ASD often exhibit hyper-serotonemia, and 5-HT levels are significantly predictive of ASD development (65). Its receptor serotonin transporter protein (SERT) was found to regulate synaptic serotonin, modulating mitochondrial copy number and mitochondrial respiratory complex gene expression in the frontal cortex and cerebellum in a sexually dimorphic manner, which could help explain the gender bias in ASD prevalence (66). Catecholamines are neurotrophic and regulatory factors, with DA primarily responsible for attention, reward motivation, motor and communication skills (67). Developmental deficits in DA neurons in the midbrain underlie the pathogenesis of ASD, with a positive correlation between low DAergic status and impaired socio-emotional and communication (68), accompanied by higher levels of hypervanillic acid (69). Effective dopamine therapy could be a potential ASD treatment. Cullin 3 (CUL3), a core component of the Cullin-RING ubiquitin E3 ligase complex, whose deficiency increases the excitability of ventral tegmental area (VTA) DA neurons, and a hyperpolarization-activated channel, HCN2, can act as a target for CUL3 in DA neurons to attenuate CUL3-induced behavioral deficits through inhibition of either D2 receptors or DA neuronal activity (70). In addition, ASD may result from decreased activity of dopamine-Β-hydroxylase (DΒH) in the hippocampus and neocortex (71), as this enzyme is regulated by a single gene, DΒH, and catalyzes conversion of DA to NE (72), which is a system that affects intelligence, memory, rewards, arousal, and attention and stress (73). Thus, perinatal DΒH gene low activity may create conditions for ASD development. At the same time, children with ASD exhibit increased tonicity and decreased phasic activity of the locus coeruleus-norepinephrine (LC-NE) system. Abnormal LC-NE activation may be associated with atypical arousal and reduced behavioral response in ASD (74).

### 3.2 Neurotransmitter receptor abnormality

Neurotransmitter receptors are proteins on neurons responsible for receiving and transmitting signals from neurotransmitters. Their abnormalities may lead to altered neuronal response to neurotransmitters, affecting neuroplasticity (75). Neuronal communication involves two trans-synaptic signaling pathways of the cell adhesion protein family, presynaptic neuregulin (Nrxn) and postsynaptic neuroligin (Nlgn), which are among the most frequently affected pathways in ASD (76). The Nrxn gene encodes presynaptic cell adhesion molecules that are important in regulating synaptic neurotransmitter release, particularly Gluergic and GABAergic transmission (77). Mutations in the Nrxn1-3 gene or disruption of its splicing program impair cognitive function in the brain, which is associated with ASD (78). Nlgn is a postsynaptic cell adhesion molecule organizing synapses. It is involved in synaptic recognition, specification, and functional maturation, and maintains E-I homeostasis in specific neural connections (79). One study found that mice prenatally exposed to valproic acid (VPA) exhibited cognitive and motor deficits in Y-maze learning, which may be due to reduced levels of proteins involved in excitatory synapse formation, such as Nlgn1 and PSD-95 (80). Among the subtypes of Nlgn, the Nlgn2 gene is often reduced in expression in severe neuropsychiatric disorders (81), Nlgn3 primarily mediates enteric neuron-neuroglial (82), and Nlgn4 is primarily involved in functioning in intelligence, social skills, sleep, and arousal (83) and impairments of all of these subtypes are associated with ASD. Additionally, GPR85, an ASD risk factor, associates with NLGN-associated postsynaptic density protein PSD-95 in brain, interfering with dendritic formation via the NLGN-PSD-95 receptor complex and causing neural excitation-inhibition imbalance, affecting learning and memory (84). These factors may be genes or signaling pathways related to ASD synaptic abnormalities, so some ASD pathogenesis may also stem from synaptic dysfunction. Moreover, choline is an important neurotransmitter with cognitive enhancement effects, and cholinergic transmission deficits may be responsible for lifelong attention deficits in ASD patients (85). Reduced M1 muscarinic cholinergic receptor activity may contribute to the ASD phenotype, whereas the use of partial M1 muscarinic receptor agonists reduces repetitive behaviors and interest restriction in a mouse model of ASD (86). The α7-nicotinic acetylcholine receptors (α7-nAChRs) have been associated with social and communication deficits in ASD, and a positively-mutated modulator (PAM) of this receptor enhances social communication behavior in a rat model of ASD (87). These findings can potentially help to identify drug therapy targets.

### 3.3 Abnormalities in neural circuits

Abnormalities in neural circuits may lead to altered brain network activity and synaptic plasticity in patients with ASD. Studies have shown that patients with ASD have abnormal electroencephalogram (EEG) activity and functional magnetic resonance imaging (fMRI) signals, which affects information transfer between neurons and impairs cognitive functions such as learning and memory (88).

Patients with ASD exhibit hyperconnectivity in localized brain areas regarding EEG functional connectivity, yet effective connectivity across hemispheres is significantly reduced (89), demonstrating hypo-connected networks and sub-optimal network characteristics. Children with ASD have increased power spectral density (PSD) fast bands (high beta and gamma), higher variability (CV), and lower complexity (MSE) compared to normally developing children (90). This suggests that in children with ASD, neural networks are more variable, less complex, and less adaptive, with reduced capacity for producing optimal responses. Research found that children with ASD have elevated relative efficacy in the δ, θ (4-8 Hz), α (8-12 Hz), Β, and γ bands, decreased θ/Β ratios in frontal regions, enhanced θ/Β ratios in Cz and Pz electrodes, and significant correlations between θ/Β and cognitive function (91). The hierarchical processing of phonemic and syllable range information (θ/γ coupling) is disrupted in children with ASD, which may account for communication difficulties in children with impaired speech reception (92). Total lobe alpha power is lower in children with ASD than in neurotypical children (93), whereas long-distance intra- and inter-hemispheric connectivity is improved after transcranial magnetic stimulation (rTMS) interventions, especially within the alpha band, while local and global network properties are greatly enhanced in the δ, θ, and α bands (94). Transcranial direct current stimulation (tDCS) holds potential in ASD treatment via plasticity modulation, acting as a novel method for motor and cognitive function enhancement to alleviate ASD symptoms (95).

fMRI is a non-invasive imaging technique used to study brain activity. Patients with ASD show high connectivity in the frontal lobe, anterior cingulate, parahippocampus, left precuneus, horn, caudate, superior temporal, and left pallidum, and low connectivity in the antero-central, left supra-frontal, left mid-orbital frontal lobe, right amygdala, and left posterior cingulate (96), suggesting abnormal neural circuitry in patients with ASD. In early ASD, reduced activation of socio-emotional verbal areas in the supratemporal cortex and the appearance of atypical connections in the visual and precuneus cortices are shown, which are closely associated with the development of social and language deficits in children with ASD (97). In addition, the lack of correlation between bilateral prefrontal cortex and cerebellar gray matter volumes and language social scores in children with ASD compared to neurotypical children may underlie language and social deficits (98). Pathological reductions in amygdala and hippocampal gray matter volume bilaterally have been associated with lower language skills (99). Underactivation of the right and left ventral striatum has been associated with lower reward seeking in children (100). In conclusion, these abnormal connections help to find biomarkers of ASD, such as EEG spectral abilities, face perception responses (101), and the brain’s default mode network (DMN) (102). The results of these studies have also helped to improve diagnostic and therapeutic methods for ASD patients. Scatter tensor imaging (DTI), functional MRI (fMRI), flexible analytic wavelet transform (FAWT) (103), and the application of artificial intelligence (104) can be new ASD detection techniques. Therefore, it is important to study non-invasive treatments such as EEG and fMRI to do a more in-depth study of specific stimuli and brainwave changes to enrich the clinical diagnosis and treatment of ASD.

## 4 Neuroinflammation and immunity

### 4.1 Neuroinflammation

Neuroinflammation affects neuronal development, connectivity, and function, including activation and proliferation of microglia and astrocytes, excessive release of pro-inflammatory cytokines and chemokines, breakdown of the blood-brain barrier, and upregulation of inflammatory signaling pathways, among others. Glial cells primarily prune synapses or respond to injury by isolating the injury site and expressing inflammatory cytokines (105). In ASD, the cell number and density of astrocytes and microglia are increased (106). Astrocytes maintain homeostasis in the brain microenvironment primarily through the uptake of ions and neurotransmitters (107). However, astrocyte aquaporin-4 (AQP4) deficiency causes synaptic dysfunction and leads to decreased social interaction and motor activity, increased anxiety, and decreased ability to recognize new objects (108). Prenatal exposure to valproic acid (VPA) produces ASD-like behavior by downregulating myeloid cell 2 (TREM2) levels and upregulating microglial cell levels, causing microglia to undergo polarization dysregulation and excessive pruning of synapses (109).

Abnormal expression of inflammatory cytokines, including interleukin-6 (IL-6), tumor necrosis factor-α (TNF-α), and interferon-γ (IFN-γ), is present in patients with ASD. Increased plasma granulocyte colony-stimulating factor (G-CSF) concentrations have also been associated with an increased risk in children diagnosed with ASD (110). Excessive release of these inflammatory cytokines causes children with ASD to exhibit higher leukocytes, monocytes, IL-1α, IL-1Β, IL-2, IL-4, IL-6, IL-8, and IL-10, macrophage (M)1 profiles, and anti-inflammatory profiles (111). Children with ASD who have gastrointestinal symptoms are often associated with increases in many innate (IL-1α, TNFα, GM-CSF, IFNα) and adaptive cytokines (IL-4, IL-13, IL-12p70) (112). IL-6 is a neurogenic cytokine that affects neuronal proliferation, synapse formation, differentiation, and migration and acts as a modulator of central neural pathways, which are important for cognitive functioning (113), and has also been associated with worsening of restricted and repetitive behaviors (114). TNF-α has homeostatic functions, such as regulating neurogenesis, myelin formation, blood-brain barrier permeability and synaptic plasticity, and mediates changes in excitatory and inhibitory neurotransmission in a concentration-dependent manner (115). Abnormalities in the TNFα/NFκB signaling axis lead to defects in neural progenitor cell proliferation and synaptic development that exhibit ASD features (116). Neurons also require physiological IFN-γ signaling to maintain CNS homeostasis, and pathological IFN-γ prolongs synaptic pathway transcript activation and impairs neural function (117). High levels of IFN-γ in children with ASD are associated with more severe symptoms, especially in the behavioral-motor, social, and language expression domains, and therefore, the level of IFN-γ derived from γδ T cells could be one of the potential candidate biomarkers for ASD (118).

Dysregulation of chemokine levels early in life hinders normal immune and neurobehavioral development, and elevated peripheral chemokine levels at birth are strongly associated with the progression of ASD (119). The expression of chemokine receptors, inflammatory mediators, and transcription factors plays a key role in neuroinflammatory diseases. A selective CXCR2 antagonist, SB33223, was able to trigger an anti-inflammatory response by down-regulating inflammatory mediators and NF-κB/Notch inflammatory signaling in mice modeled with ASD (120), which could be a novel target for therapy. In addition, in individuals with ASD, the blood-brain barrier is impaired and permeability is increased (121), and high sensitivity C-reactive protein (hsCRP), prolactin (PRL), and serum reactive oxygen metabolites (ROM) are markedly elevated (122), accompanied by mitochondrial dysfunction and neuronal oxidative stress (123). Based on the neuroinflammatory theory, peripheral activation of immune-inflammatory pathways may lead to neuroinflammation and mitochondrial dysfunction in the CNS, resulting in abnormalities in transsynaptic transmission and brain neurodevelopment (124). Sphingosine-1-phosphate (S1P), neuron-specific enolase (NSE) (125), glial fibrillary acidic protein (GFAP), and neurofilament (Nfl) (126) are expected to be used as biomarkers for the diagnosis of ASD. Notably, some studies have found that prebiotics and probiotics have sufficient anti-inflammatory and antioxidant properties to be considered useful dietary components to help prevent ASD (127).

### 4.2 Neuroimmunity

Children with ASD are associated with immune abnormalities in the brain and periphery, including inflammatory diseases and autoimmunity, which can occur in affected individuals and mothers, disrupting neurodevelopmental function. Abnormal activation and functional alterations of immune cells are present in patients with ASD, including activation of microglia, macrophages, and abnormal expression of T cells. Disruption of glial cell function in ASD may affect normal neurotransmitter metabolism, synaptogenesis, and cause brain inflammation (128). Deficiency of TMEM59 in microglia impairs their synaptic phagocytosis by destabilizing the C1q receptor CD93, leading to enhanced excitatory neurotransmission and increased dendritic spine density (129). Maternal immune activation (MIA) alters the microglia phenotype in the brains of fetal and neonatal mouse offspring, resulting in a large increase in the number of microglia, as well as excessive proliferation of neural progenitor cells in the subventricular zone (SVZ), resulting in neurological abnormalities (130). Minocycline inhibits microglia activation and may alleviate ASD-like behavior and improve neurogenesis (131). The gut microbiome is also an important regulator of microglia and ASD-like social behavior (132). Microbial interventions, including diet, probiotics, antibiotics, and fecal transplants, as well as immunomodulatory therapies such as cytokine blockade during preconception, pregnancy, and the postpartum are currently improving neurodevelopment, behavioral patterns, and gastrointestinal health in individuals with autism (133).

It has been found that patients with ASD have a significant decrease in regulatory B cells, CD4+ lymphocytes with regulatory T lymphocytes (Tregs) and an increase in Th17 lymphocytes (134). T cell apoptosis plays a crucial role in the pathogenesis of inflammatory diseases. Whereas in T lymphocytes from subjects with ASD, dysregulation of thioredoxin reductase-1 (TrxR1)/thioredoxin-1 (Trx1) redox reactions, which is associated with reduced Bcl2 expression and increased apoptosis, leads to reduced T cell survival in ASD patients (135). Depletion of CD4 T cells results in memory deficits that are exacerbated over time (136). Photobiomodulatory treatment (PBMT) exerts beneficial neuromodulatory effects by activating the JAK2/STAT4/STAT5 signaling pathway to promote IFN-γ/IL-10 expression in non-substantial CD4 T cells and induces improved brain microenvironmental conditions and alleviates cognitive deficits in a mouse model (137). Moreover, neonatal immune profiles vary by sex, and their cytokine and chemokine concentrations are sex-differentiated and associated with neurodevelopmental outcomes (138).

Villin-1 (vil1) may be a key pathway in MIA-induced ASD, and knockdown of the fatty acid-binding protein 2 (fabp2) gene in zebrafish rescues social behavioral deficits in MIA offspring (139). MIA is also gender-specific in influencing social communication behavior patterns, with males showing impaired preference for olfaction in social stimuli, and females showing aggression in social encounters and reacting more strongly to somatosensory stimuli (140). Immune dysregulation by maternal autoantibodies (aAbs) also disrupts metabolic signaling and induces neuroanatomical alterations in the brain of the offspring, demonstrating a reduction in verbal expression and marked deficits in social play behavior (141).

## 5 Interactions of genetics, environment, neural pathways, neuroinflammation and neuroimmunity

Although the genetic environment, neural pathways, neuroinflammation, and immunity can all contribute to ASD development, the individual factors often do not occur in isolation from one another, but rather sequentially or concurrently and interact with one another to create ASD’s complex pathology. An adverse gestational environment can affect fetal maturation and development, inducing abnormal gene expression or mutations. Prenatal exposure to clinically relevant progestins in pregnant dams decreased estrogen receptor Β expression in the offspring’s amygdala, manifesting as autism-like behavior (142). Notably, Haddad et al. found that both MIA and Cntnap2 gene deficiency resulted in similar ASD-like behavioral deficits, but that both independently and synergistically exacerbated ASD-like symptoms in model rats when acted upon together (143). The opposite result was obtained by Kim et al. Knockout of the Cntnap2 gene and prenatal exposure to valproic acid, as two risk factors for both genetic and environmental factors, acted together in a model mouse, which was found to have improved social deficits, which may be related to the correction of abnormal glutamatergic neuron excitability (144). It is clear that the effects of multiple factors superimposed on ASD are bi-directionally modulated compared to single factors. More than 800 genes and dozens of genetic syndromes have been found to be associated with ASD (1). Mutations in genes associated with neural formation and development can directly affect neural function and produce corresponding ASD symptoms. For example, Ash1L haploinsufficiency leads to defective synaptic pruning of neurons in the brain, triggering ASD-related behavioral deficits, and these abnormal behaviors are mediated by cortical circuits (24). Neuroinflammation and neuroimmunity also play an important role in this, Engrailed-2 knockout mice have an increased density of astrocytes in the brain and induce neuroinflammatory and neurodegenerative changes (145). ASD patients have a significant overexpression of histaminergic system-related genes in the brain and chronic neuroinflammation, which may be due to the mechanism that histaminergic system affects neuroinflammation by regulating microglial cell activation, cytokine release, and migration to influence neuroinflammation (146). MIA during pregnancy exacerbates ASD-like symptoms in Shank3-deficient mice and is accompanied by the upregulation of postsynaptic densitin, impairing the normal functioning of neural pathways (147). MIA may also increase the risk of ASD by dysregulating aspects of fetal brain gene expression, highlighted by transcriptional dysregulation of mTOR and EIF4E, which affect a variety of key neural developmental functions (148).

**Figure 3:**
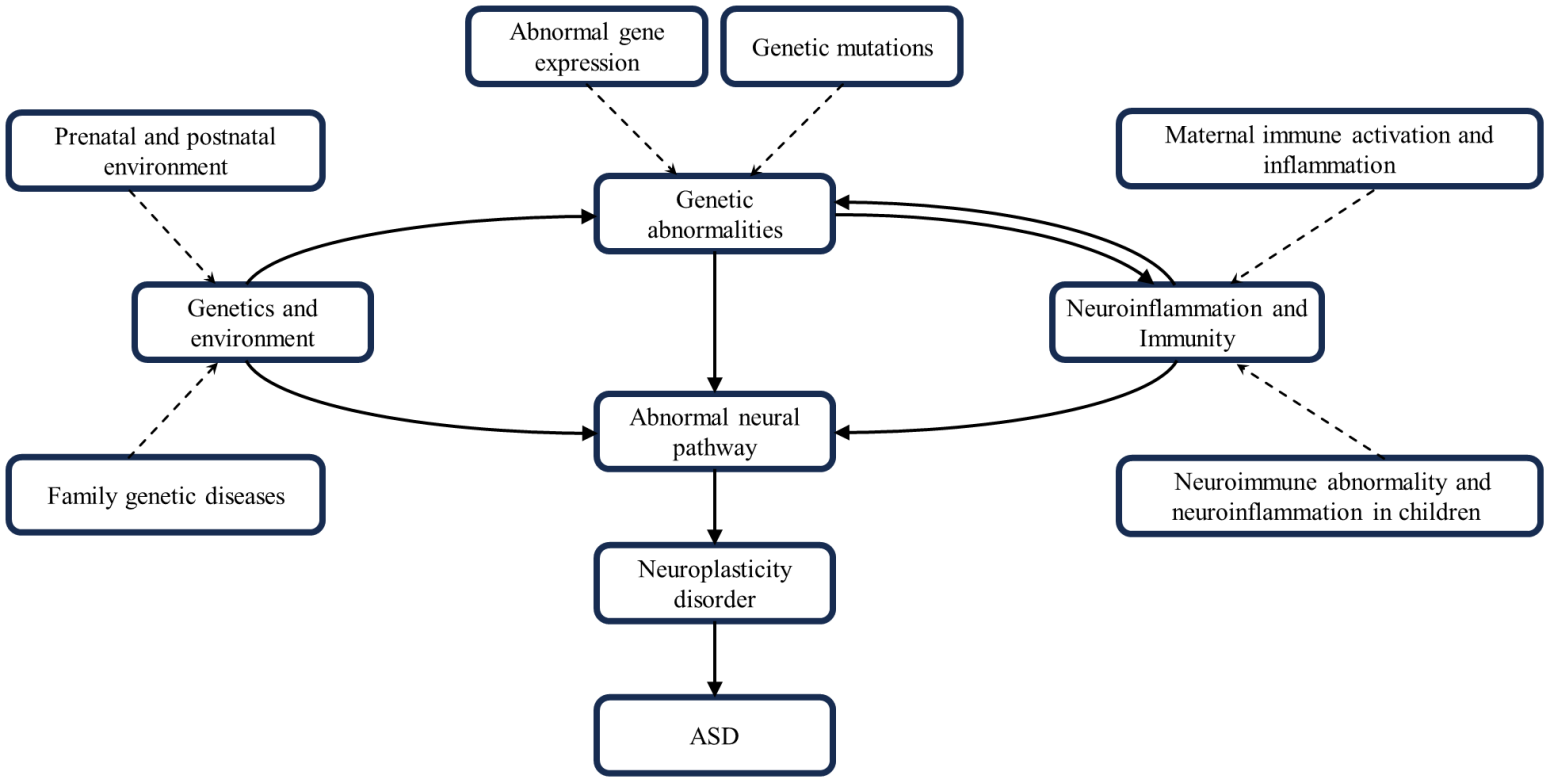
Interactions of genetics, environment, neural pathways, neuroinflammation and neuroimmunity. These factors combine to contribute to the development of ASD.

In summary, ASD development is a complex process involving multiple factors and pathways. Genetic factors provide the basis for ASD development; environmental factors may trigger or exacerbate these genetic predispositions. Neuroinflammatory and neuroimmune responses occur throughout. They all work together to influence brain development and function by disrupting neural pathway function and impairing neuroplasticity, leading to the characteristic symptoms of ASD. The interactions and cumulative effects of these factors form a complex network in the pathogenesis of ASD (**Figure 3**).

## 6 Conclusion

Analysis of current literature sources on ASD mechanisms demonstrates their polygenic, complex, multifactorial nature and association with brain development and dysfunction. Most researchers agree that the development of ASD is not necessarily determined by a single gene, but rather is the result of a combination of mutations in many genes, with a certain degree of heritability. However, ASD is not a genetic disorder, especially in the early stages of development, and many potential risk factors, such as poor maternal pregnancy and childbirth, may trigger mutations that produce ASD symptoms. Severity and symptom variability can vary. Genetic and environmental factors can activate the nervous system in a number of ways, shifting neurodevelopment toward the autistic type. These factors usually play a role in the early stages of neurological formation such as the perinatal and early postnatal periods. Thus, we can explore abnormal neuroplasticity during a critical period of neurological development. However, the mechanisms by which these factors influence the development of abnormal neuroplasticity in ASD remain to be further investigated and demonstrated.

Altered excitatory-inhibitory neurotransmission plays a key role in ASD and is an important component of the molecular mechanisms of abnormal plasticity. Abnormalities in neurotransmitters such as glutamate, gamma-aminobutyric acid, and dopamine can dysregulate neural homeostasis and function. Abnormalities in neurotransmitter receptors and neural pathways involved in signaling of neural pathways can also result in neurodevelopmental dysfunction. However, all of these factors can be considered as complementary diagnostic criteria for ASD without a harmonized and highly selective and sensitive biosensor, while more new biomarkers for ASD are yet to be discovered. These targets can provide broader avenues and ideas for ASD drug therapy development and research.

Although the exact pathophysiology of ASD is not well defined, there is growing evidence to support a role for neuroinflammation and immune dysregulation. Glial cells, cytokines, and chemokines can induce neuroinflammation, which in turn affects synaptic plasticity. Understanding the mechanisms of neuron-glia interactions on synapse formation and maturation will help develop new therapeutic targets for neurodevelopmental disorders. The microbial-gut-brain axis involving the microbial-immune-neuronal domain is also a hotspot for neuroinflammatory research. Furthermore, although animal models support a causal relationship between MIA and ASD development, their validity remains to be explored. Therefore, a neuroplasticity perspective to unravel potential neuromolecular mechanisms targeting ASD could serve as a novel approach for developing ASD interventions, diagnoses, and treatments.

## Author contributions

ZC: Writing – review & editing, Writing – original draft, Investigation. XW: Writing – review & editing, Software. SZ: Writing – review & editing, Investigation. FH: Writing – review & editing, Supervision, Methodology, Conceptualization.
